# Male Nurses.—II

**Published:** 1890-10-25

**Authors:** 


### Passing Topics.

### MALE NURSES.—II.

Admitting that male nurses are wanted, and even under the present *régime* recourse must not infrequently be had to them, it follows that opportunities should be supplied to render them capable and trustworthy. It need not be feared that men will ever supplant women in the field of nursing, but that means should be claimed for them to qualify for tending certain classes of sufferers of their own sex is a demand which commends itself as eminently reasonable.

If we consider the capacity of men for nursing, the verdict of all experienced persons would probably be that, while suitability for this occupation is less common in men than in women, male nurses, when good, are difficult to improve upon, and instances might be given where approval has been wrung even from those prejudiced against them. We need not go back to the middle ages in search of illustration. It is within the experience of most people who have been placed in positions to judge, that some of the tenderest and most devoted services have been rendered by men to the sick. In the Arctic seas, in the jungles of India, in the deserts of the Soudan, in camp and field, in countless journeyings beyond the limits of civilization, man has ministered to suffering man with unsurpassable kindness and devotion, while the record of every marching regiment is full of deeds of gentleness rendered by comrade to comrade. By all means let women remain the "ministering angels," but we need not deny to the rougher sex those attributes of humanity which go towards the making of a good nurse.

Mr. Stanley, in his latest book, furnishes yet another example of a natural capacity for nursing even among the savages of Central Africa, and gives a touching account of a husband's care of a wife sorely sick of a repulsive malady Among the children of the poor in our own land the question of who shall be the children's nurse is usually determined by the accident of birth, and the eldest boy or girl occupies the position indifferently, with not less of credit in the one case than the other.

So much for natural aptitude; but in order to become skilful and capable of acting up to the requirements of physicians and surgeons in the present enlightened times, training is needed, and training is precisely what men cannot now obtain, nor will they be enabled to obtain it until some strong current of feeling is set in motion. The improvement in nursing generally, dates from the time when the light of day was let in upon the shortcomings and enormities of the old system, and as surely as the significance of such facts as we referred to in our last issue, but purposely refrained from dwelling upon, comes to be fully apprehended, will an irresistible demand arise for facilities to train male nurses. The Hamilton Association, founded curiously enough by a lady, must not remain passive under rebuff if it would be the pioneer. Let it take heart of grace and agitate until it does get an opening. The individual or the organization which succeeds in this matter will deserve well of the community, and especially of our women nurses, who to inevitable hardship, and much that is unavoidably painful must now add duties, the bare mention of which revolts, and which, not upon one ground, but upon all grounds, it is very undesirable they should perform. Of course the reception of male probationers would necessitate proper provision for them. If men were added to an establishment without it, the result could not fail to be disastrous; but given suitable arrangements, careful selection of candidates, and sensible government, we see no reason to doubt that an ample supply of male nurses would be in time forthcoming, altogether unlike "the men from the street" who are now the butts, and not unreasonably, of so much feminine satire.

